# Acacetin improves endothelial dysfunction and aortic fibrosis in insulin-resistant SHR rats by estrogen receptors

**DOI:** 10.1007/s11033-020-05746-3

**Published:** 2020-09-06

**Authors:** Yaxin Wei, Peipei Yuan, Qi Zhang, Yang Fu, Ying Hou, Liyuan Gao, Xiaoke Zheng, Weisheng Feng

**Affiliations:** Henan University of Chinese Medicine, Zhengzhou, 450046 China

**Keywords:** Acacetin, Endothelial dysfunction, Aortic fibrosis, Estrogen receptors

## Abstract

The aim of the work was to investigate the effects of acacetin on endothelial dysfunction and aortic fibrosis in insulin-resistant SHR rats and explore its mechanism. Seven-week-old male spontaneously hypertensive rats (SHR) were selected to establish a rat model of hypertension with insulin resistance induced by 10% fructose. The nuclear factor kappa B p65 (NF-κB p65) and Collagen I were observed by Immunohistochemistry. Immunofluorescence was used to observe estrogen receptor-alpha (ERα), estrogen receptor-beta (ERβ), and G protein-coupled receptor 30 (GPR30). Western blotting was used to detect interleukin (IL-1β), Arginase 2 (ARG2), Nostrin, endothelial nitric oxide synthase (eNOS), TGF-β, Smad3, ERK pathway proteins such as p-c-Raf, p-MEK1/2, p-ERK, ERK, p-P90RSK and p-MSK1. We found that acacetin did have an improvement on endothelial dysfunction and fibrosis. Meanwhile, it was also found to have a significant effect on the level of estrogen in this model by accident. Then, the experiment of uterine weight gain in mice confirmed that acacetin had a certain estrogen-like effect in vivo and played its role through the estrogen receptors pathway. In vitro experience HUVEC cells were stimulated with 30 mM/L glucose and 100 mM/L NaCl for 24 h to establish the endothelial cell injury model. HUVEC cells were treated with 1 μM/L estrogen receptors antagonist (ICI 182780) for 30 min before administration. Cell experiments showed that acacetin could reduce the apoptosis of HUVEC cells, the levels of inflammatory cytokines and the expression of TGF-β, Collagen I and Smad3 in endothelial cell injury model. After treatment with ICI 182780, the improvement of acacetin was significantly reversed. The results showed that acacetin relieved endothelial dysfunction and reduced the aortic fibrosis in insulin-resistant SHR rats by reducing the release of inflammatory factors and improving vasodilatory function through estrogen signaling pathway.

## Introduction

Hypertension is a major public health problem in the world, endangering people's life and health. According to the recently published *China cardiovascular disease news 2018*, there are currently about 245 million patients with hypertension in China. The prevalence rate in adults is as high as 23.2% and the incidence of insulin resistance in patients with hypertension is as high as 30% to 50% [1]. Patients with hypertension and insulin resistance have a significantly increased risk of cardiovascular disease. In diabetics, for example, every 5 mmHg increase in systolic or diastolic blood pressure is associated with a 20–30% increase in the risk of cardiovascular disease [2].

Cardiovascular diseases have a high incidence of morbidity and mortality, one of the main causes is vascular endothelial dysfunction, that is, endothelial-dependent vascular diastolic dysfunction, resulting in reduced vascular compliance and reduced blood flow [3]. Vascular endothelial dysfunction is manifested in impaired endothelial barrier function, resulting in the muscle layer being damaged by various growth factors or inflammatory factors. On the other hand, it is manifested in impaired vasodilation, vascular remodeling, imbalance of coagulation and anticoagulant mechanism, etc.[4].

Endothelial cells (EC) are monolayer epithelial cells distributed on the inner surface of vessels, which not only act as a barrier between blood circulation and surrounding tissues, but also regulate vascular tension, blood flow velocity and platelet function actively [5]. Dysfunction of vascular endothelial cells leads to vascular inflammation, which increases the secretion of inflammatory factors such as TNF-α, IL-1β, NF-κB p65 and so on. It is also closely related to arginase, a urea circulating enzyme that competes with nitric oxide synthase (NOS) for its common substrate L-arginine, reducing the production of NO. NO can stimulate vascular smooth muscle relaxation, dilate blood vessels and regulate blood flow. When the bioavailability of NO decreases, endothelial function of blood vessels will be damaged [6]. Arginase exists in two subtypes: arginase 1(ARG1) is found in the cytoplasm and most expressed in the liver, while ARG2, mainly in the mitochondria, is mainly distributed in the kidney. Both subtypes exist in vascular endothelial cells and smooth muscle cells and can be up-regulated by high glucose and reactive oxygen species [7]. Studies have shown that over-activation of ARG2 in aorta and decrease of eNOS can lead to endothelial dysfunction [8].

Vascular endothelial dysfunction is also associated with vascular fibrosis, which leads to thickening of the vessel wall, decreasing vascular compliance and increasing stiffness. TGF-β/Smad3 signal transduction plays a central role in the pathogenesis of fibrosis [9] and the mechanism of pathological changes in vascular fibrosis is related to Collagen I deposition of extracellular matrix and proliferation of fibroblast cells [10]. Abnormal thickening of blood vessels and fibrosis caused by collagen deposition is the major cause of arteriosclerosis and an independent cardiovascular risk factor.

Studies have shown that women generally have a lower risk of cardiovascular disease than men, but this protective effect disappears after menopause, meaning the importance of estrogen [11]. Many experimental studies in cardiovascular disease have also confirmed the efficacy of estrogen therapy, including lowering blood pressure, inhibiting atherosclerosis, endothelial dysfunction, and reducing myocardial ischemic injury [12, 13]. Part of the mechanism of estrogen's cardioprotective effect is its direct effect on the vascular wall, mainly on vascular endothelial cells and vascular smooth muscle cells. Vascular injury response is the key link of cardiovascular disease. The main pathological changes are vascular endothelial injury and smooth muscle proliferation and migration [14]. On the other hand, estrogen can also directly affect insulin and glucose homeostasis, improve body fat distribution and reduce insulin resistance and inflammation. Studies have shown that increased insulin resistance during puberty in men is associated with decreased estrogen. Compared with premenopausal women, men have more visceral adipose tissue and higher insulin resistance, which may be related to lower levels of endogenous estrogen [15–17]. Estrogen (E2) is a natural hormone secreted by the mature follicles of the ovary. The E2 can bind to a variety of receptors, such as the classic estrogen receptors ERα, ERβ and GPR30. Estrogen receptors signaling not only regulates gene transcription in the nucleus through “the classical nuclear pathway”, but also plays a role by regulating the binding of ligands to estrogen receptors. Cells respond very quickly to estrogen through enzyme-mediated activation of membrane-related estrogen, known as the "non-nuclear" ER signal [18]. In endothelial cells, estrogen rapidly activates eNOS in 15 to 30 min via a "non-nuclear" signaling pathway [19].

Estrogen regulates a variety of cell functions through nuclear and non-nuclear signaling pathways or ligand-independent signaling pathways, which can inhibit proliferation of cardiac fibroblasts, hypertrophy and apoptosis of cardiomyocytes, proliferation of vascular endothelial cells and activation of eNOS signal. In our previous studies, it was found that a variety of compounds isolated from traditional Chinese medicine can promote the proliferation of MCF-7 cells and the expression of estrogen receptor [20–22].

Modern pharmacological studies have shown that chrysanthemum has anti-inflammatory, vasodilating, lowering blood pressure, improving insulin resistance and other pharmacological activities [23, 24]. As a traditional Chinese medicine with significant improvement in cardiovascular diseases, chrysanthemum was studied in our laboratory for the chemical constituents of flavonoids [25]. Through the preliminary experiments, acacetin was screened out showing an improvement on the endothelial cell injury model. Therefore, in this study, fructose-fed SHR was used to establish the model of hypertension combined with insulin resistance, so as to the mechanism of improving endothelial dysfunction and aortic fibrosis was studied.

In order to explore the vascular protective mechanism of acacetin, estrogen receptors expression in aortic tissues was detected in animal experiments. It was accidentally found that acacetin could improve the expression of estrogen receptors in the aortic tissues of insulin-resistant SHR rats. Therefore, it was confirmed whether acacetin had estrogen activity through the uterine weight gain experiment in mice and further verified whether the mechanism of acacetin's vascular protective effect was related to estrogen receptors in vitro cell experiments. In vitro experiments using high sugar, high salt stimulate HUVEC injury model, selection of estrogen receptors antagonist (ICI182780), verify whether acacetin can activate the expression of estrogen receptors, reduce the release of inflammatory factors, improve vasodilatation function, alleviate the endothelial dysfunction, thus improving insulin resistance SHR rats aorta fibrosis.

## Materials and methods

### Reagents and antibodies

The antibodies against IL-1β (ab9722), TM (ab187075), TGF-β (ab92486), Collagen I (ab34710), ERα (ab32063), ERβ (ab3576), GPR30 (ab39742) were purchased from Abcam (UK). The antibodies against eNOS (#32027), p-ERK (#4377), p-c-Raf (#9911), p-MEK1/2 (#9911), p-P90RSK (#9911), p-MSK1 (#9911) were from Cell Signaling Technology (USA). The antibodies against NF-κB p65 (10745-1-AP), ARG-2 (14825-1-AP), Nostrin (20116-1AP), Smad3 (25494-1-AP), ERK (16443-1-AP) were from Proteintach Group Company (USA). The antibodiy against (Ser612) p-IRS-1 (BS5084) was from Bioworld (Germany). The antibodiy against β-tubulin (AC008) was from ABclonal (China). IRDye® 680 RD Goat anti-Rabbit (C80911-11) and IRDye® 800CW Goat anti-Mouse (C80816-16) were purchased from LI-COR company (USA). The RIPA Lysis Buffer (R0013B) and the BCA Protein Assay Kit (PC0020) were purchased from Solarbio Science & Technology Co., Ltd. (Beijing, China). TGX™ FastCast™ Acrylamide Kit (#161-0173) was purchased from Bio-Rad Company (USA). PageRuler™ Prestained Protein Ladder (26617) was from Thermo Fisher Scientific (USA). Iodine [^125^I] Insulin Radioimmunoassay kit (F01-INS) was purchased from Beijing north institute of biotechnology (China). Rat Von Willebrand Factor (vWF, E-EL-R1079c), Rat P-Selectin (E-EL-R0828c), Rat tumor necrosis factor α (TNF-α,E-EL-R2856c) and Mouse Estradiol (E2, E-EL-0150c) elisa kit were purchased from Elabscience Biotechnology Co., Ltd. (Wuhan, China). The NO (A013-2) kit was purchased from Nanjing Jiancheng biological engineering institute (China). Annexin-v apoptosis assay kit with PE (8086787) was purchased from BD biosciences (USA).

### Drug

Chrysanthemum was collected from Wuzhi County Jiaozuo City and identified by professor Suiqing Chen of Henan University of Traditional Chinese medicine as the dried flower head of chrysanthemum (*Chrysanthemum morifolium* Ramat.“*Xiaohuaiju*”cv.nov.). The specimens (2017-JH) were kept in the laboratory of traditional Chinese medicine chemistry, school of pharmacy, Henan University of traditional Chinese medicine.

Chrysanthemum was weighed 11.2 kg and extract twice with 50% acetone. It was combined with low temperature decompression filtrate concentration to get the total extract. The total extract was dispersed in water and extracted by systematic solvent method. Petroleum ether, ethyl acetate, n-butanol and water parts were obtained in turn. 352.0 g of the dried ethyl acetate was weighed, dissolved in ethyl acetate, mixed with silica gel and dried by evaporation. Silicagel column chromatography was performed. Dichloromethane and methanol were eluted by gradient at a ratio of 50:1. After drying, Toyopearl HW-40 was added to the solution, followed by elution with water and different proportion of aqueous methanol gradient, and the elution part was repeatedly eluded with sephadexLH-20, ODS, Silica gel, MCL Gel CHP-20 and other columns, and then separated and purified by the method of preparation of high performance liquid phase and recrystzation to obtain acacetin 12.3 g (AC).

Fructose (A100226-0005) was purchased from Diamond (China). Acacetin (B20627) was purchased from Yuanye Biological Co., Ltd. (Shanghai, China). Valsartan (H200505508) was purchased from Huanglong pharmaceutical co., Ltd. (Hainan, China). Estradiol valerate (H20160679) was purchased from Bayer healthcare co., Ltd. (Beijing, China). Fulvestrant, ICI 182780 (ICI) was purchased from the MCE Company (USA).

### Grouping and administration of animals

Seven-week-old male Wistar Kyoto Rats (WKY) and spontaneously hypertensive Rats (SHR), weighing 180–200 g, were purchased from Charles River (Beijing, China). Animal license number is SCXK (Jing) 2016-0006. WKY and SHR rats with a body weight of 180–200 g were randomly divided into 5 groups. WKY rats were set as the normal control group and given distilled water for free drinking and 1 mL/100 g distilled water for ig. SHR rats were randomly divided into four groups and given 10% fructose water for free drinking. The four groups were the model group SHR(F) (1 mL/100 g distilled water, ig), the low-dose acacetin group SHR(F)+AC-L (25 mg/kg acacetin,ig), the high-dose acacetin group SHR(F) + AC-H (50 mg/kg acacetin,ig), the positive group SHR(F) + Y (30 mg/kg valsartan, ig).

Female Kunming mouse of clean grade just weaned at 21 days old, weighing 9-12 g, were purchased from the same company. Animal license number is SCXK (Jing) 2016-0011. The mouse were divided into four groups based on weight balance and randomization, and were given the drug for seven days. The normal group (NC) was given 1 mL/100 g distilled water daily, the positive control group (Y) was given 0.33 mg/kg estradiol valerate every other day, and the administration group was given AC-L (35 mg/kg) and AC-H (70 mg/kg).

### Detection of Systolic blood pressure (SBP) and insulin resistance index (HOMA-IR)

SBP of the rats was measured by non-invasive caudal arterial blood pressure meter (Techman, China) every week. The blood was collected from orbital venous plexus of the rats once every 2 weeks, and fasting blood glucose was measured by glucometer (On·Call EZII, China). The blood samples were centrifuged at 4 °C, 3000 *g* for 20 min and the supernatant was taken as serum. The fasting insulin level was measured by radioimmunoassay and the insulin resistance index was calculated. The experimental results showed that acacetin treatment could significantly reduce the SBP and HOMA-IR of the rats after six weeks, so 10% chloral hydrate (0.3 mL/100 g) was intraperitoneally injected into the rats for anaesthesia and anatomical sampling. Aorta was randomly selected from each group and fixed with 4% paraformaldehyde. Other fresh tissues were rapidly quenched in liquid nitrogen and immediately transferred to the − 80 °C refrigerator for storage.

### Hematoxylin and eosin staining

Fresh aortic tissue was fixed with 4% paraformaldehyde for more than 24 h, then the tissue was removed, repaired and leveled and put in a dehydration box for dehydration with gradient alcohol in turn in the dehydrator. Paraffin sections were dewaxed to water, the sections were stained with hematoxylin and eosin (G1005, Servicebio, China) dye, then dehydrated and sealed. After microscopic examination (Nikon Eclipse E100, Japan), the image was collected and analyzed (Nikon DS-U3, Japan).

### Detection of biochemical indicators

The levels of vWF, P-Selectin and TNF-α in rat serum were determined by ELISA. Nitrate reductase method was used to detect the level of NO in rat serum. The level of E2 in mouse serum was determined by ELISA.

### Western blotting

Rat aortic tissue was taken from the refrigerator at – 80 °C and the total aortic protein was extracted from RIPA lysate. The supernatant was centrifuged at 12000 rpm at 4 °C for 5 min. The total protein was quantified with the BCA protein concentration assay kit. The total protein was prepared into 1 × sample using 5 × SDS protein electrophoresis loading buffer solution (WB-0091). The total protein was boiled in a boiling water bath for 10 min and centrifuge at 12,000 rpm at 4 °C for 10 min. Each group was subjected to sodium dodecyl sulfate—polyacrylamide gel electrophoresis (SDS-PAGE) at a concentration of 30 μg/10 μL. The target bands were transferred to polyvinylidene fluoride (PVDF) film (ISEQ00010, Millipore, USA). After being sealed with 5% BSA (9048-46-8, Genview, USA) for 1 h, specific antibodies of the antigen to be tested were added such as IL-1β, TM, ARG2, Nostrin, eNOS, TGF-β, Smad3, p-c-Raf, p-MEK1/2, p-ERK, ERK, p-P90RSK and p-MSK1. The room temperature shaker was incubated for 30 min, overnight at 4 °C and the next day for 30 min, then rinsed with 0.2% Tween20 phosphate buffer solution (PBST) for 5 times, 5 min each. The corresponding fluorescent secondary antibodies were added and incubated in darkness at room temperature for 1 h, then rinsed with 0.2%PBST for 3 times, 5 min each. The imaging was scanned with Odyssey dual-color infrared fluorescence imaging system and the data was quantified (LI-COR, USA).

### Immunohistochemistry

Paraffin sections were dewaxed to water and aortic tissue sections were placed in citric acid antigen repair buffer (G1202, servicebio, China) for antigen repair in microwave oven. After natural cooling, the slides were placed in PBS and washed on the shaker for 3 times, 5 min each time. Sections were placed in 3% hydrogen peroxide solution, incubated in dark for 25 min at room temperature and washed in PBS 3 times for 5 min each. The tissue was uniformly covered by 3%BSA and sealed at room temperature for 30 min. Gently shake off the sealing solution and add a primary antibody to NF-κB p65, Collagen I and (Ser612) p-IRS-1. The sections were laid flat in a wet box and incubated overnight at 4 °C. After washing with PBS for 3 times each time for 5 min, the corresponding secondary antibody was added, followed by incubation at room temperature for 50 min, then washing with PBS for 3 times each time for 5 min. After the sections were slightly shaken dry, the freshly prepared DAB color solution (K5007, DAKO, Germany) was dripped and the section was rinsed with running water to stop the color development. The nuclei were restained with hematoxylin, dehydrated and sealed and the images were collected for analysis. Image Pro Plus (IPP) software was used for Image analysis and semi-quantitative processing of immunohistochemical results.

## Immunofluorescence

Paraffin sections were dewaxed to water and aortic tissue sections were placed in a box (G1206, servicebio, China) filled with EDTA antigen repair buffer for antigen repair in microwave oven. After natural cooling, the slides were placed in PBS and washed by shaking on the shaker for 3 times, 5 min each time. After the sections were slightly shaken dry, a tissue pen was used to draw a circle around the tissue to prevent the antibody from flowing away. The spontaneous fluorescence quenching agent was added into the circle for 5 min and then washed by running water for 10 min. After adding BSA, the sealing solution was shaken off and incubated for 30 min. Then, primary antibodies ERα, ERβ and GPR30 were added to the sections and laid flat in a wet box at 4 °C for overnight incubation. The second antibody was added and incubated in dark room for 50 min. DAPI dye (G1012,servicebio,China) was incubated in dark room for 10 min. Anti-fluorescence quenching and sealing tablets (G1401,servicebio,China) were used for microscopic examination after sealing. IPP software was used for Image analysis and semi-quantitative processing of immunohistochemical results.

### Cell culture

HUVEC cells purchased from the Shanghai Cell Bank of the Chinese Academy of Sciences were cultured in DMEM/LOW GLUCOSE 1X (PE2190108, Proteineasy, China) containing 10% fetal bovine serum (11011-8611, Tianhang, China). The medium was added with triple antibodies containing 10 kU/mL penicillin, 10 mg/mL streptomycin and 5 mg/mL gentamicin. The cells were cultured in a constant temperature incubator (Thermo, USA) at 37 °C and 5% CO_2_. The experiment was conducted when the cells grew to 70–80% in logarithmic growth period.

### Flow cytometry of HUVEC cells

HUVEC cells were inoculated on 6-well plates at the density of 10 × 10^4^/mL. The experiment was divided into NC group, M group, low dose group (M-AC-1) 1 μM/L, medium dose group (M-AC-5) 5 μM/L and high dose group (M-AC-10) 10 μM/L. After the drug intervention, the cells were digested with EDTA-free trypsin, centrifuged at 1500 rpm for 8 min and the supernatant was discarded. The cells were washed twice with cold PBS and re-suspended in 1 × binding buffer. The solution was transfered 100 µL. It was added 5 µL of PE Annexin V and 5 µL 7-AAD, then incubated for 15 min at RT (25 °C) in the dark. The 1 × binding buffer was added to the suspension, which was transferred into the instrument to detect the cells by flow cytometry (FACS Aria III; BD Biosciences, USA).

### Detection of NO in HUVEC cells

In vitro cell experiments, HUVEC cells were divided into normal group (NC), model group (M), acacetin treatment group (AC) for 10 μM/L and acacetin plus estrogen receptors antagonist group (AC + ICI). In the group of M, 30 mM/L glucose and 100 mM/L NaCl were added to stimulate cells for 24 h. In the group of AC, acacetin was added. In the group of AC + ICI, 1 μM/L estrogen receptors antagonist was used to stimulate cells for 30 min before administration. The level of NO in the supernatant of HUVEC cells was detected by nitrate reductase.

### In-cell-western of HUVEC cells

HUVEC cells were inoculated in 96-well plates (1247971, Thermo, USA) with a density of 4 × 10^4^ /mL, with the specific grouping and drug intervention method as described in the detection of NO. After the drug intervention, the medium was discarded and 3.7% formaldehyde was added to fix the cells for 20 min. 0.1% Triton was permeated for 5 times for 5 min each. 5% BSA room temperature shaker was closed for 1.5 h. Specific antibodies, such as IL-1β, eNOS, TGF-β, Smad3 and Collagen I were added to dilute the antigens to be tested. After incubation at 4 °C for 18 h, 0.1%PBST was cleaned 5 times for 5 min each. After incubation for 1 h with corresponding fluorescent secondary antibody and avoiding light, 0.1%PBST was cleaned three times for 5 min each. The imaging was scanned and quantified in Odyssey dual-color infrared fluorescence imaging system.

### Western blotting of HUVEC cells

HUVEC cells were inoculated in petri dishes at a density of 2 × 10^5^/mL, with the specific grouping and drug intervention methods as described in the detection of NO. After the drug intervention, the cells were washed with PBS and 500 mL RIPA lysate was added to extract cell proteins. The levels of NF-κB p65, p-ERK, ERK and ARG2 in the cells were detected.

### Statistical analysis

SPSS 20.0 software was used for statistical analysis of the data, which was expressed as $\overline{x}$ ± s. One-way ANOVA was used to compare the differences between groups. *P* < 0.05 or *P* < 0.01 were considered to be statistically significant.

## Results

### Effects of acacetin on SBP and HOMA-IR in SHR rats with insulin resistance

**Fig. 1:**
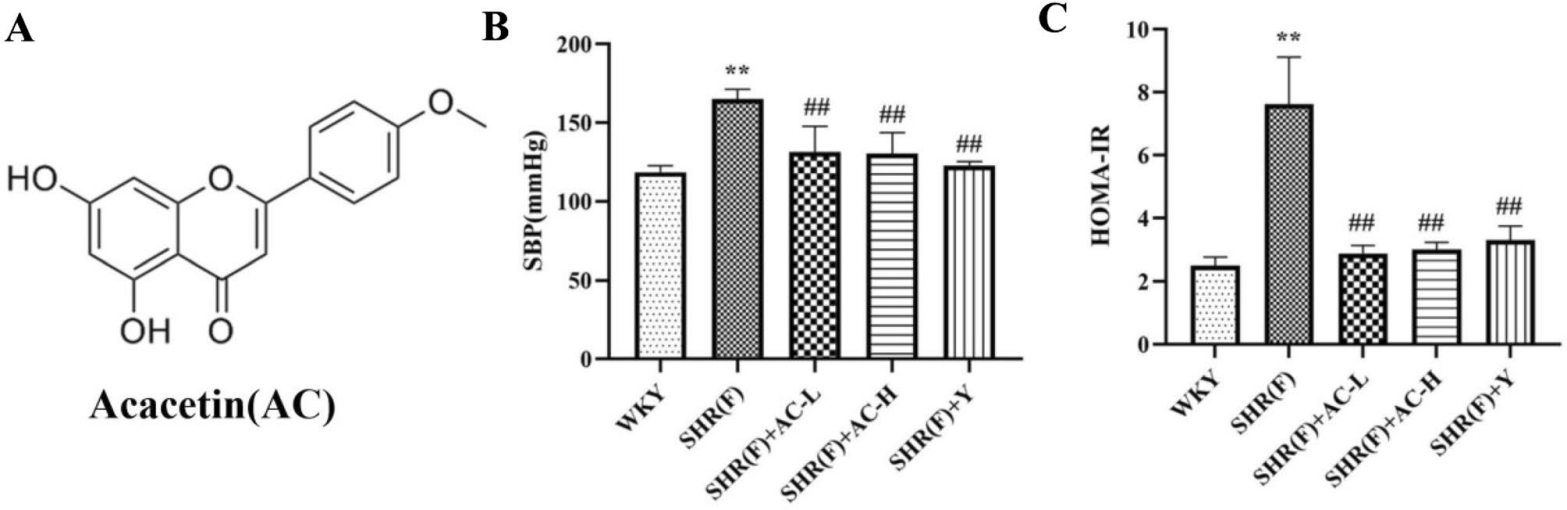
Effects of acacetin on SBP and HOMA-IR in SHR rats with insulin resistance. **a** The structural formula of acacetin. **b** The level of SBP. **c** The level of HOMA-IR. Values are expressed as the mean ± SD of 6 samples. ^#^*P* < 0.05, ^##^*P* < 0.01 vs. SHR(F) group. ^*^P < 0.05, ^**^P < 0.01 vs. WKY group

Compared with the WKY group, SBP and HOMA-IR of the rats in the SHR(F) group were significantly increased (*P* < 0.01), suggesting that both hypertension and insulin resistance existed in the model group. The low and high dose of acacetin significantly reduced the levels of both groups (*P* < 0.01), suggesting that acacetin could improve the status of hypertension and insulin resistance in SHR(F) rats (Fig. 1).

### Effects of acacetin on aortic structure and function in SHR rats with insulin resistance

**Fig. 2:**
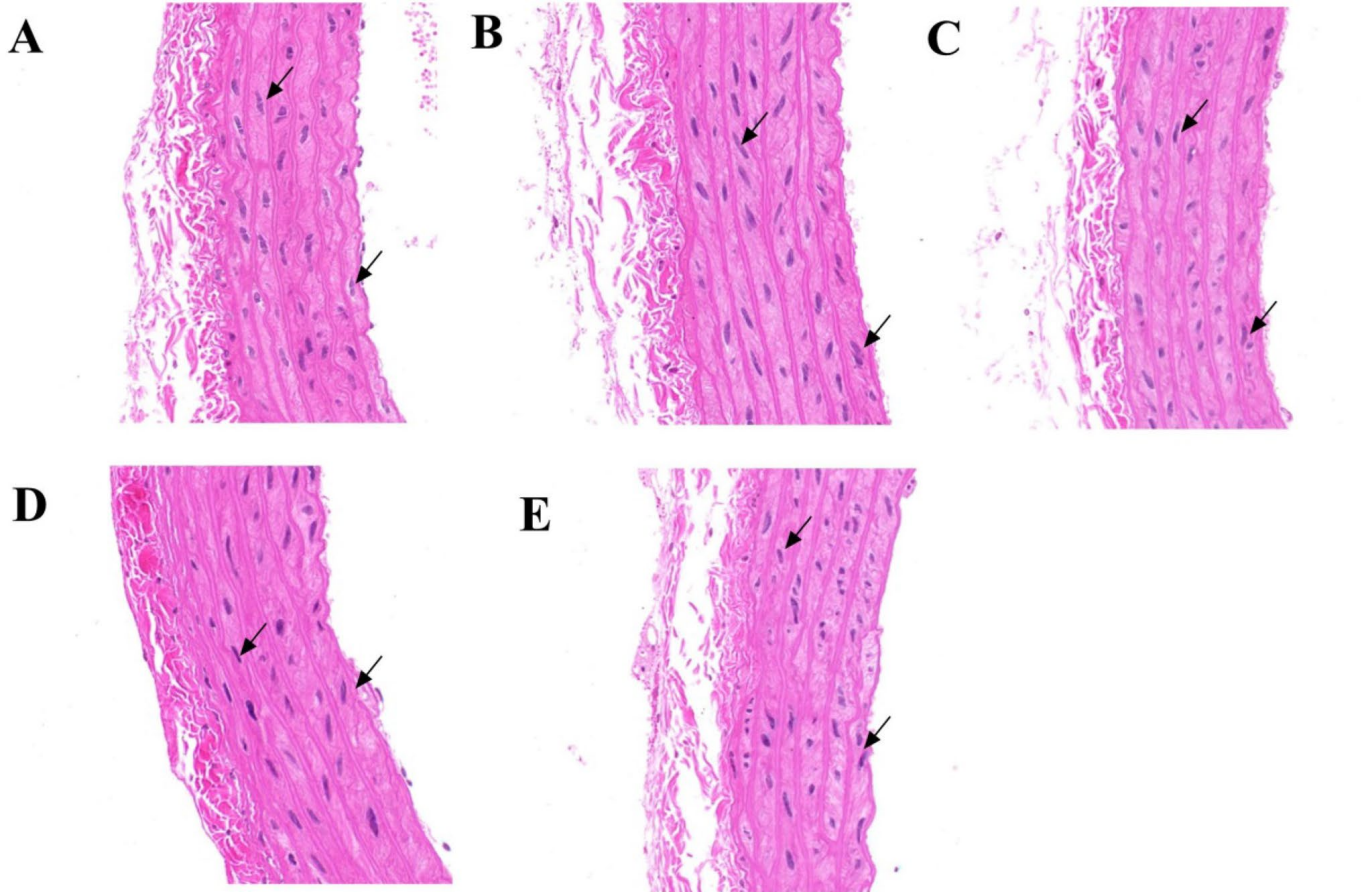
Effects of acacetin on aortic structure and function in SHR rats with insulin resistance. **a** WKY group, **b** SHR(F) group, **c** SHR(F) + AC-L group, **d** SHR(F) + AC-H group, **e** SHR(F) + Y group. The magnification of HE staining results were 400X. The arrows point to the smooth muscle and endothelial nuclei

Observation under light microscope showed that the aortic wall of normal group rats was of moderate thickness, vascular endothelial cells and smooth muscle cells were evenly distributed, elastic fibers were clear and complete, arranged in a ring, and the outer membrane was thin layer of loose connective tissue. Compared with the WKY group, the aortic wall of the rats in the SHR(F) group was significantly thickened, vascular endothelial cells and smooth muscle cells were enlarged and the arrangement was disordered, indicating morphological damage of the aortic vascular wall in the rats in the model group. After administration, aortic wall thickening was improved and endothelial cells and smooth muscle cells returned to normal, suggesting that acacetin could improve the morphological damage of aortic wall in rats (Fig. 2).

### Effects of acacetin on vascular endothelial injury in SHR rats with insulin resistance

**Fig. 3:**
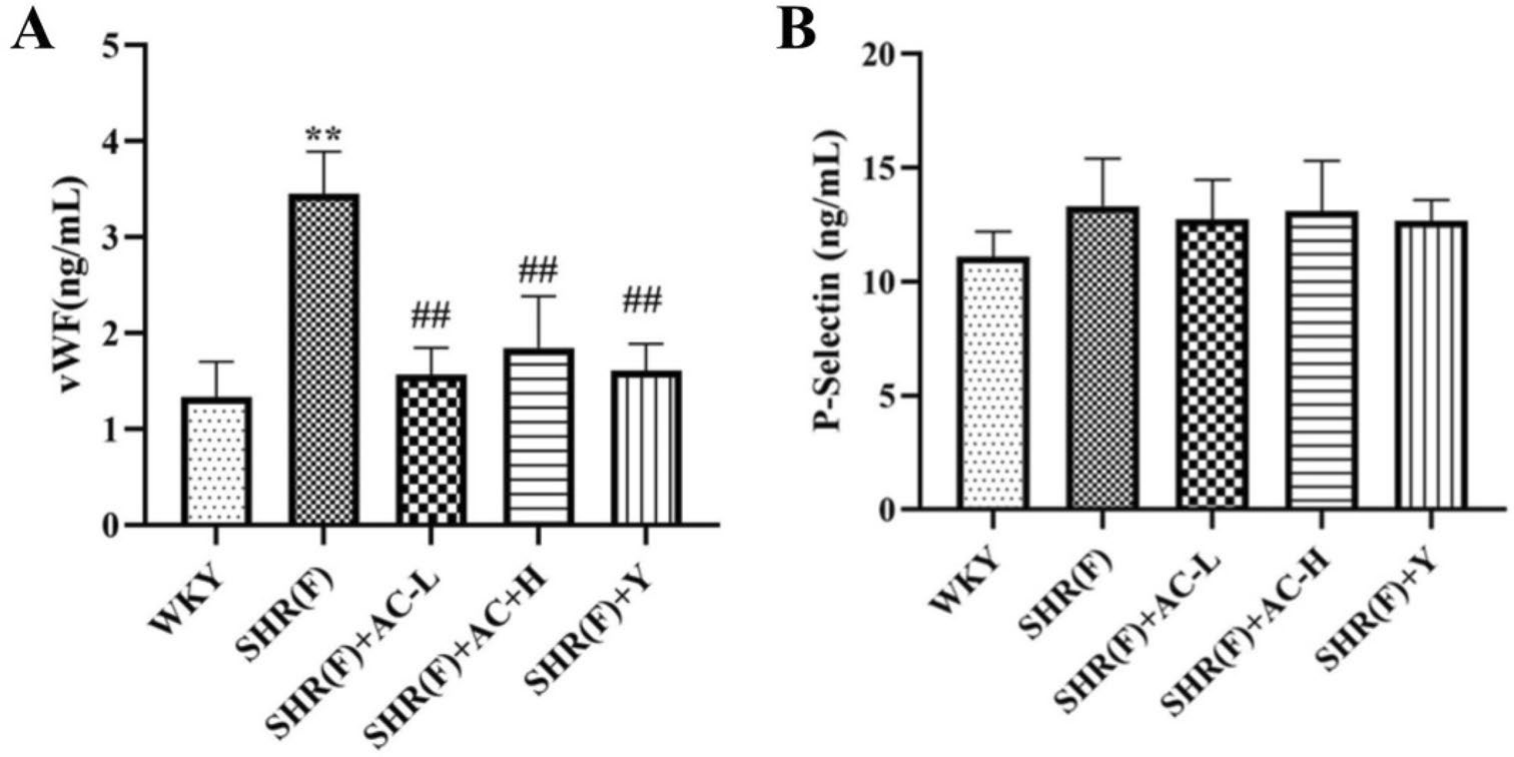
Effects of acacetin on vascular endothelial injury in SHR rats with insulin resistance. **a** The level of vWF in serum was detected by ELISA. **b** The level of P-Selectin was detected by ELISA. Values are expressed as the mean ± SD of 6 samples. ^#^*P* < 0.05, ^##^*P* < 0.01 vs. SHR(F) group. ^*^P < 0.05, ^**^P < 0.01 vs. WKY group

Compared with the WKY group, the vWF in the SHR(F) group was significantly increased (*P* < 0.01). The P-Selectin showed an upward trend, indicating vascular endothelial injury and dysfunction in the model group. Both the low and high dose groups of acacetin observably reduced the level of vWF (*P* < 0.01) and decreased the expression of P-Selectin, suggesting that acacetin can improve vascular endothelial dysfunction in SHR(F) rats (Fig. 3).

### Effects of acacetin on (Ser612) p-IRS-1 in SHR rats with insulin resistance

**Fig. 4:**
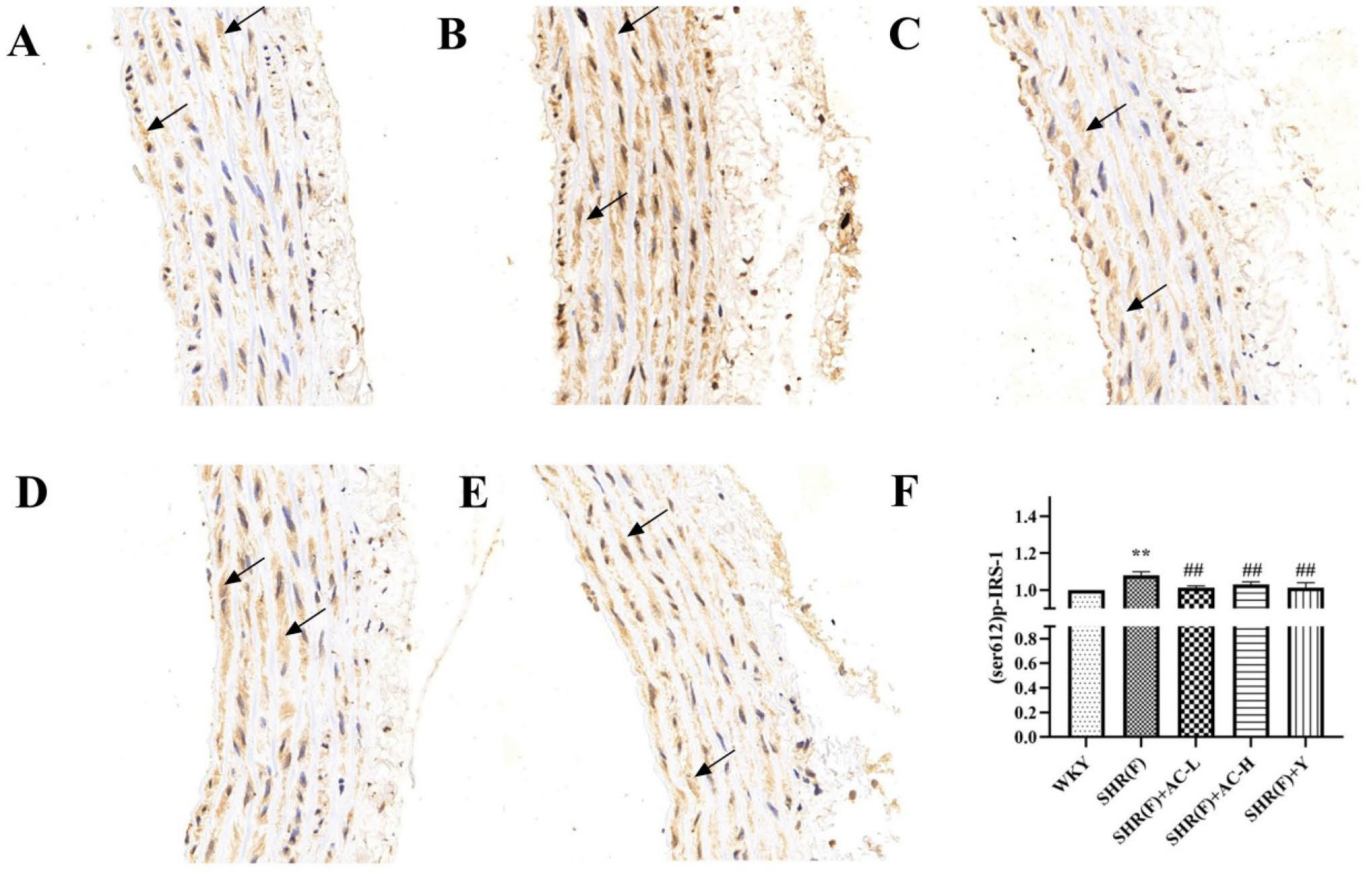
Effects of acacetin on (Ser612) p-IRS-1 in SHR rats with insulin resistance. The level of (Ser612) p-IRS-1 in rat aortic tissue was detected by immunohistochemistry. The magnification of the immunohistochemical results were 400X. The arrows indicate the position of (Ser612) p-IRS-1 positive expression. **a** WKY group, **b** SHR(F) group, **c** SHR(F) + AC-L group, **d** SHR(F) + AC-H group, **e** SHR(F) + Y group. **f** The quantitative results of immunohistochemistry. Values are expressed as the mean ± SD of 6 samples. ^#^*P* < 0.05, ^##^*P* < 0.01 vs. SHR(F) group. ^*^P < 0.05, ^**^P < 0.01 vs. WKY group

Immunohistochemical results showed that the nucleus was blue and (Ser612) p-IRS-1 were brownish yellow. Compared with the WKY group, the (Ser612) p-IRS-1 content in the SHR(F) group was significantly increased (*P* < 0.01), suggesting that Ser612 phosphorylation of insulin receptors occurred in the model group rats. The low and high dose of acacetin observably reduced the level of (Ser612) p-IRS-1 (*P* < 0.01), indicating that the improvement of insulin resistance in SHR(F) rats by acacetin may be related to the inhibition of insulin receptor phosphorylation at Ser612 (Fig. 4).

### Effects of acacetin on inflammation-related indicators in SHR rats with insulin resistance

**Fig. 5:**
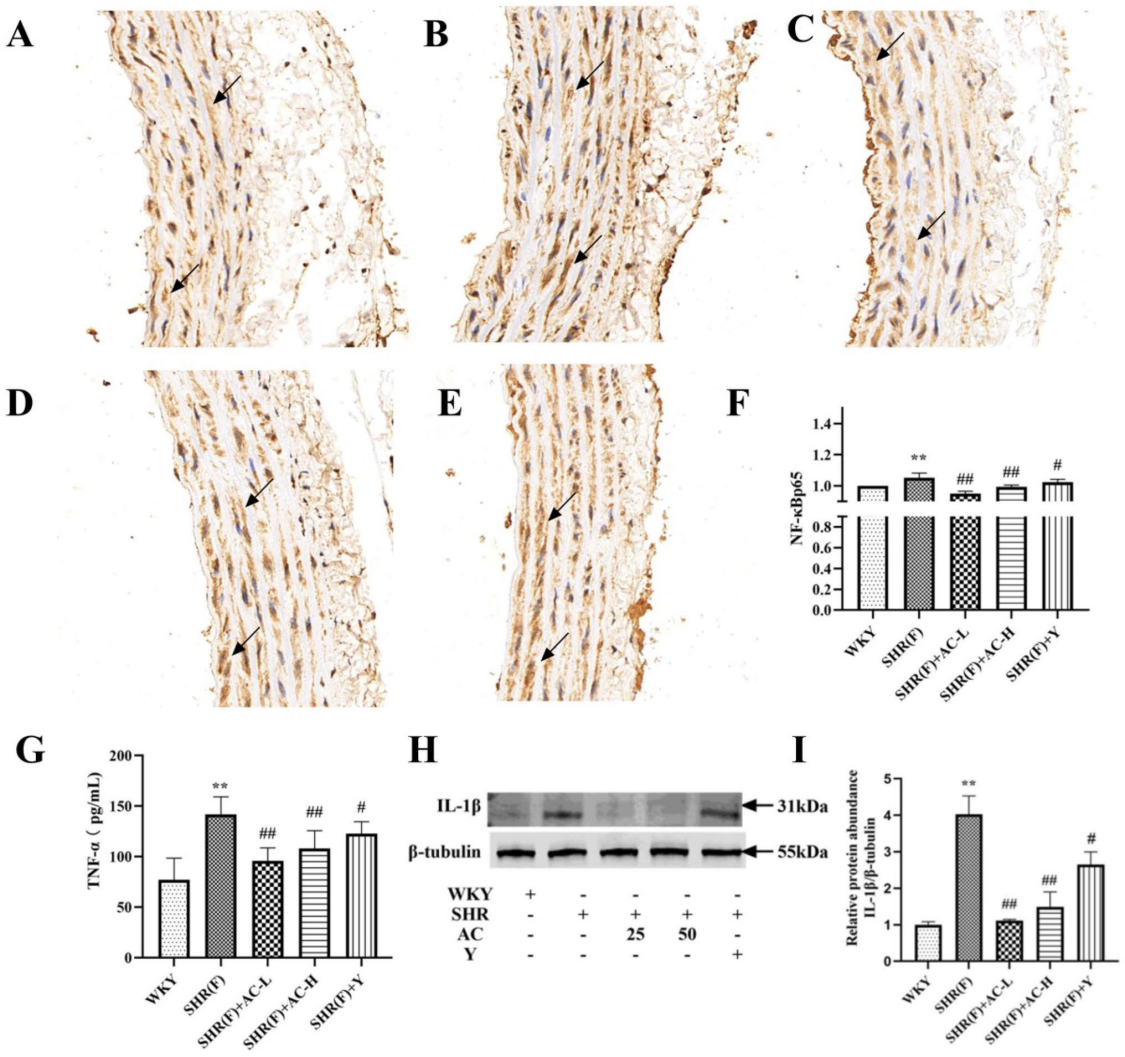
Effects of acacetin on inflammation-related indicators in SHR rats with insulin resistance. (A-F): The level of NF-κB p65 in rat aortic tissues were detected by immunohistochemistry. The magnification of the immunohistochemical results were 400X. The arrows indicate the position of NF-κB p65 positive expression. **a** WKY group, **b** SHR(F) group, **c** SHR(F) + AC-L group, **d** SHR(F) + AC-H group, **e** SHR(F) + Y group. **f** The quantitative results of immunohistochemistry. **g** The level of TNF-α in serum was detected by ELISA. **h** The level of IL-1β was detected by Western blotting. **i** The quantitative results of Western blotting. Values are expressed as the mean ± SD of 6 samples. ^#^*P* < 0.05, ^##^*P* < 0.01 vs. SHR(F) group. ^*^P < 0.05, ^**^P < 0.01 vs. WKY group

Immunohistochemical results showed that the nucleus was blue and NF-κB p65 was brownish yellow. Compared with the WKY group, the level of inflammatory cytokines TNF-α and IL-1β in the SHR(F) group were significantly increased (*P* < 0.01), suggesting an inflammatory response in the model group. The low and high dose of acacetin markedly reduced the levels of all three groups (*P* < 0.01), manifesting that acacetin can reduce inflammatory response (Fig. 5).

### Effects of acacetin on related indicators of vasodilatory function in SHR rats with insulin resistance

Compared with WKY group, there was no significant change in TM in SHR(F) group. The level of ARG2 and Nostrin were markedly increased (*P* < 0.05 or 0.01), while the levels of eNOS and NO were significantly decreased (*P* < 0.05 or 0.01). The results showed that the aortic diastolic function of the model group was impaired.

**Fig. 6:**
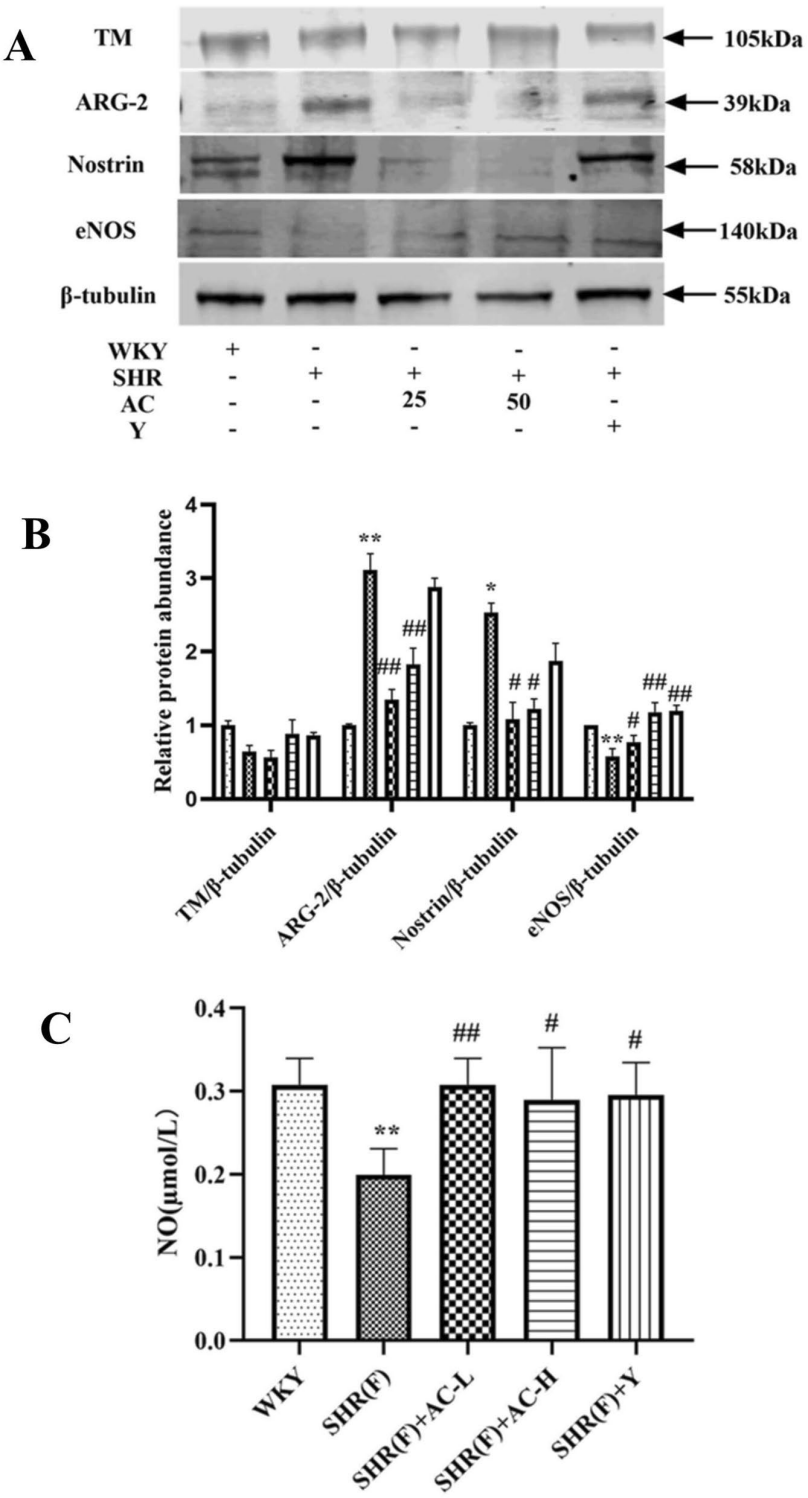
Effects of acacetin on related indicators of vasodilatory function in SHR rats with insulin resistance. **a** The level of TM, ARG2, Nostrin and eNOS in rat aortic tissues were detected by Western Blotting. **b** The quantitative results of Western Blotting. **c** Nitric acid reductase was used to detect NO in rat serum. Values are expressed as the mean ± SD of 3 samples. ^#^*P* < 0.05, ^##^*P* < 0.01 vs. SHR(F) group. ^*^P < 0.05, ^**^P < 0.01 vs. WKY group

The level of ARG2 and Nostrin were significantly decreased (*P* < 0.05 or 0.01), and the level of eNOS and NO were observably increased (*P* < 0.05 or 0.01) in the low and high dose groups of acacetin. It was manifested that acacetin could prefect vasodilatory function by promoting the expression of NO (Fig. 6).

### Effects of acacetin on vascular fibrosis in SHR rats with insulin resistance

**Fig. 7:**
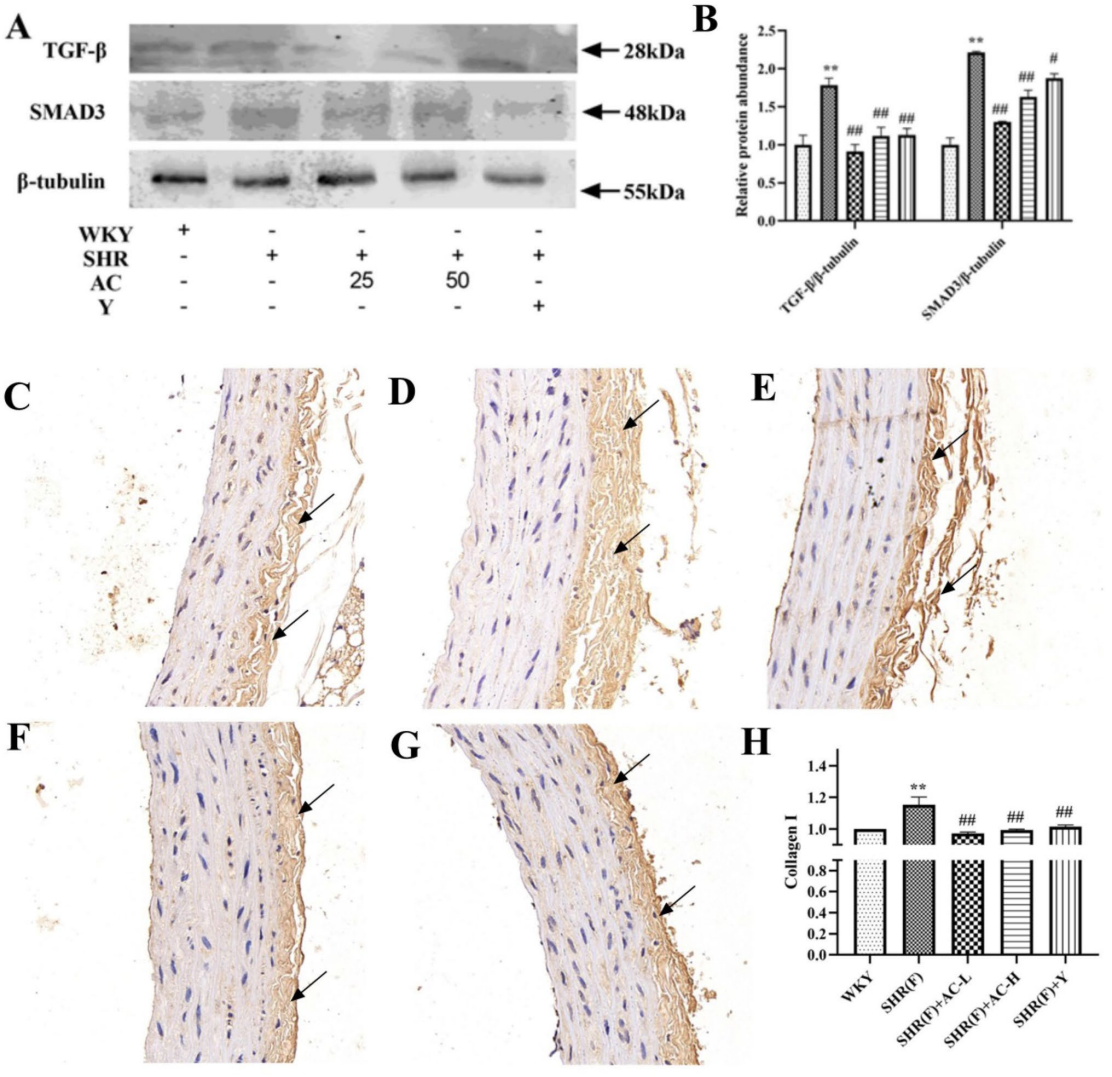
Effects of acacetin on vascular fibrosis in SHR rats with insulin resistance. **a** Western Blotting was used to detect TGF-β and Smad3 in rat aortic tissue. **b** The quantitative results of Western Blotting. **C–h** The level of Collagen I in rat aortic tissues were detected by immunohistochemistry. The magnification of the immunohistochemical results were 400X. The arrows indicate the position of Collagen I positive expression. **c** WKY group, **d** SHR(F) group, **e** SHR(F) + AC-L group, **f** SHR(F) + AC-H group, **g** SHR(F) + Y group. **h** The quantitative results of immunohistochemistry. Values are expressed as the mean ± SD of 3 samples. ^#^*P* < 0.05, ^##^*P* < 0.01 vs. SHR(F) group. ^*^P < 0.05, ^**^P < 0.01 vs. WKY group

Immunohistochemical results showed that the nucleus was blue and Collagen I was brown-yellow. Collagen I was mainly distributed in the outer membrane of the aorta with obvious hyperplasia and disordered arrangement. Compared with the WKY group, the level of TGF-β and Smad3 in the SHR(F) group were significantly increased (*P* < *0*.01), suggesting vascular fibrosis in the model group. The levels of TGF-β, Smad3 and Collagen I were dramatically decreased in the low and high dose groups (*P* < 0.01), suggesting that acacetin can improve the status of SHR(F) rats' vascular fibrosis (Fig. 7).

### Effect of acacetin on ERK signaling pathway in SHR rats with insulin resistance

**Fig. 8:**
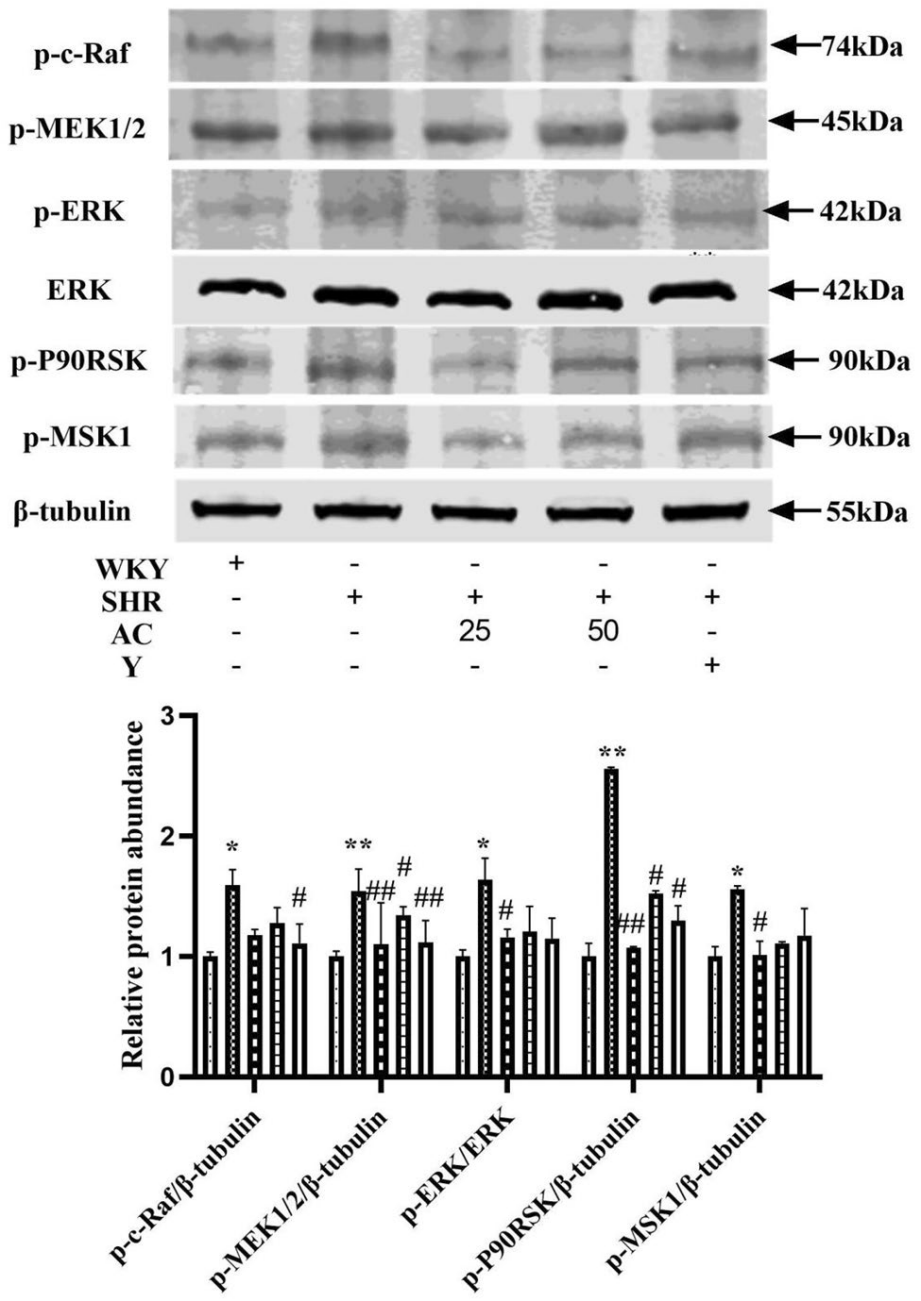
Effect of acacetin on ERK signaling pathway in SHR rats with insulin resistance. Values are expressed as the mean ± SD of 3 samples. ^#^*P* < 0.05, ^##^*P* < 0.01 vs. SHR(F) group. ^*^P < 0.05, ^**^P < 0.01 vs. WKY group

Compared with WKY group, p-c-Raf, p-MEK1/2, p-ERK/ERK, p-P90RSK and p-MSK1 in SHR(F) group were significantly increased (*P* < 0.05 or 0.01). The low and high dose groups of acacetin memorably reduced their level (*P* < 0.05 or 0.01). It was indicated that acacetin could improve the pathological state of SHR(F) rats, which might be related to the activation of ERK pathway (Fig. 8).

### Effects of acacetin on estrogen receptors in SHR rats with insulin resistance

**Fig. 9:**
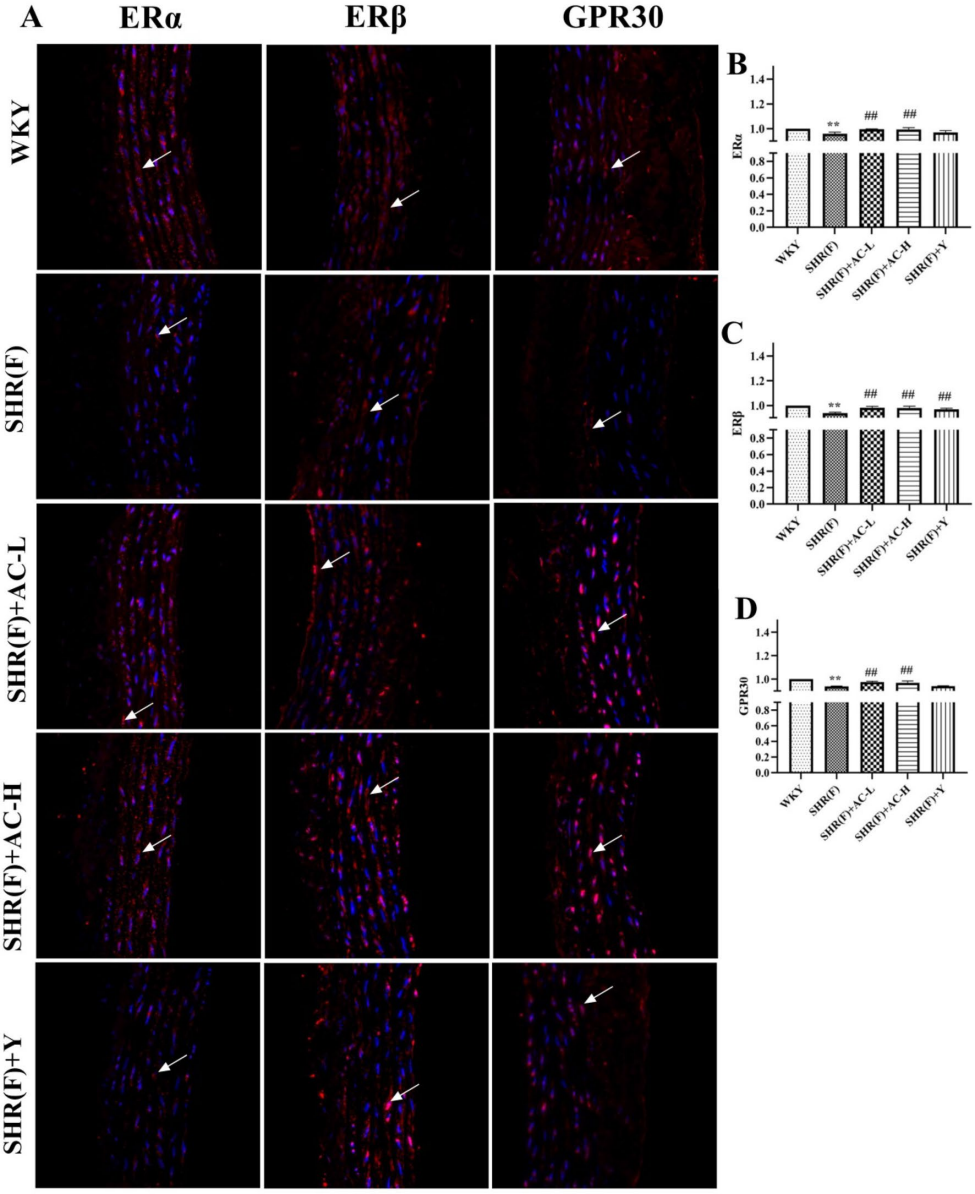
Effects of acacetin on estrogen receptors in SHR rats with insulin resistance. **a** The levels of ERα, ERβ and GPR30 in rat aortic tissues was detected by immunofluorescence. The magnification of the immunohistochemical results were 400X. The arrows indicate the position of ERα, ERβ and GPR30 positive expression. **b** The quantitative results of ERα. **c** The quantitative results of ERβ. **d** The quantitative results of GPR30. Values are expressed as the mean ± SD of 6 samples. ^#^*P* < 0.05, ^##^*P* < 0.01 vs. SHR(F) group. ^*^P < 0.05, ^**^P < 0.01 vs. WKY group

Immunofluorescence showed that the nucleus was blue and the estrogen receptors was red. Compared with WKY group, the levels of ERα, ERβ and GPR30 in SHR(F) group were significantly decreased (*P* < 0.01). The low and high dose of acacetin observably reduced the level of estrogen receptor (*P* < 0.01), indicating that acacetin can activate estrogen receptors (Fig. 9).

### Effects of acacetin on uterine coefficient, E2, ERα, ERβ and GPR30 in mouse

**Fig. 10:**
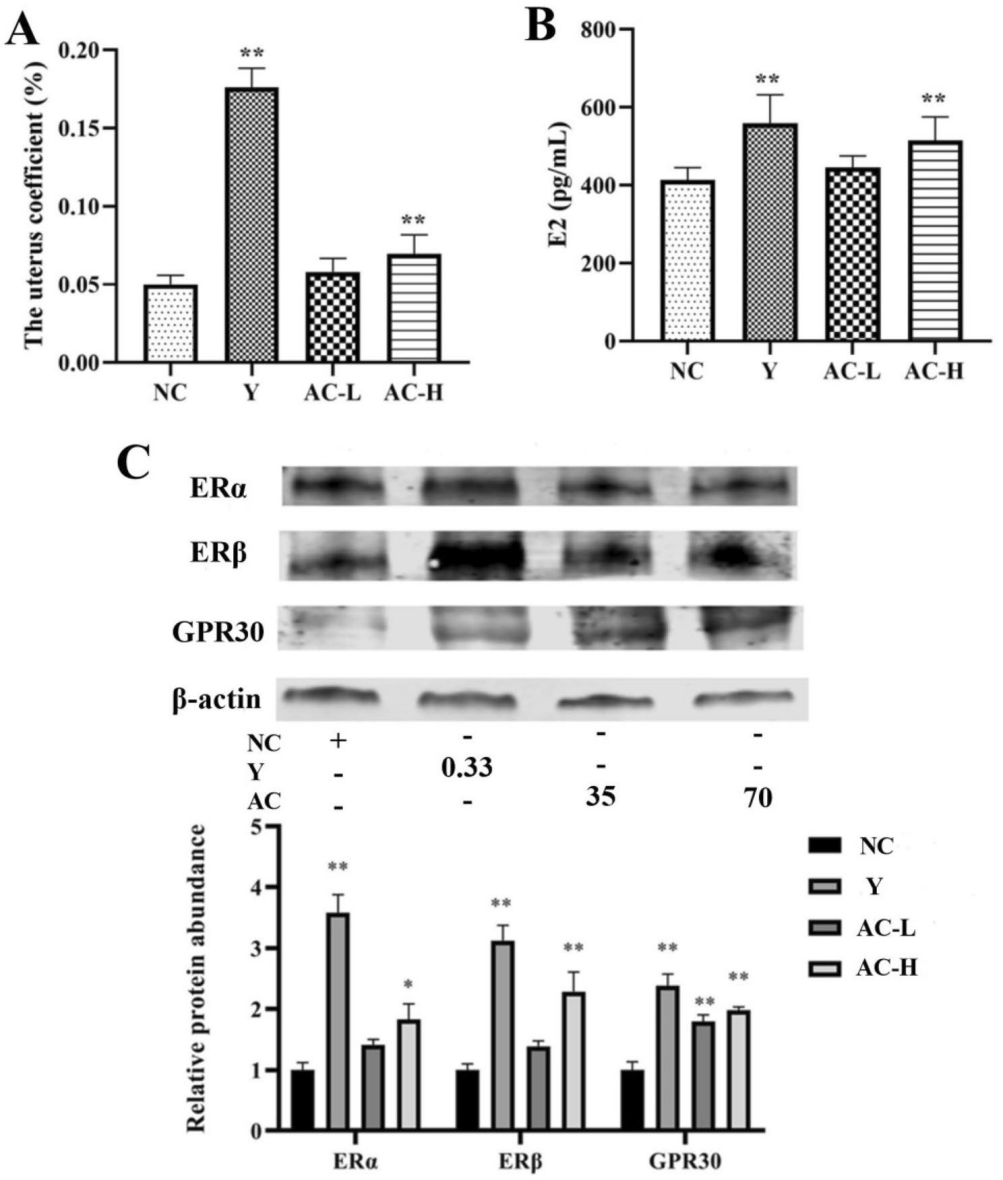
Effects of acacetin on uterine coefficient, E2, ERα, ERβ and GPR30 in mouse. **a** The effect of acacetin on mouse uterine coefficient. **b** The level of E2 in mouse serum detected by ELISA. **c** The levels of ERα, ERβ and GPR30 in mouse uterine tissue detected by Western blotting. Values are expressed as the mean ± SD of 3 samples. ^*^P < 0.05, ^**^P < 0.01 vs. NC group

Compared with the NC group, acacetin significantly increased the uterine coefficient of immature female mouse (*P* < 0.01), suggesting that acacetin has estrogen-like effects in vivo. Compared with the NC group, acacetin markedly increased the level of serum E2 in mouse (*P* < 0.01), manifesting that the estrogen-like effect of acacetin in vivo may be related to the promotion of E2 secretion in mouse. Compared with the NC group, acacetin significantly increased the expression of estrogen receptors ERα, ERβ and GPR30 in the mouse uterus (*P* < 0.05 or 0.01). These results showed that estrogen-like effects of acacetin in vivo may also be associated with increased estrogen receptor’ level in mouse (Fig. 10).

### Effect of acacetin on apoptosis of HUVEC cells

**Fig. 11:**
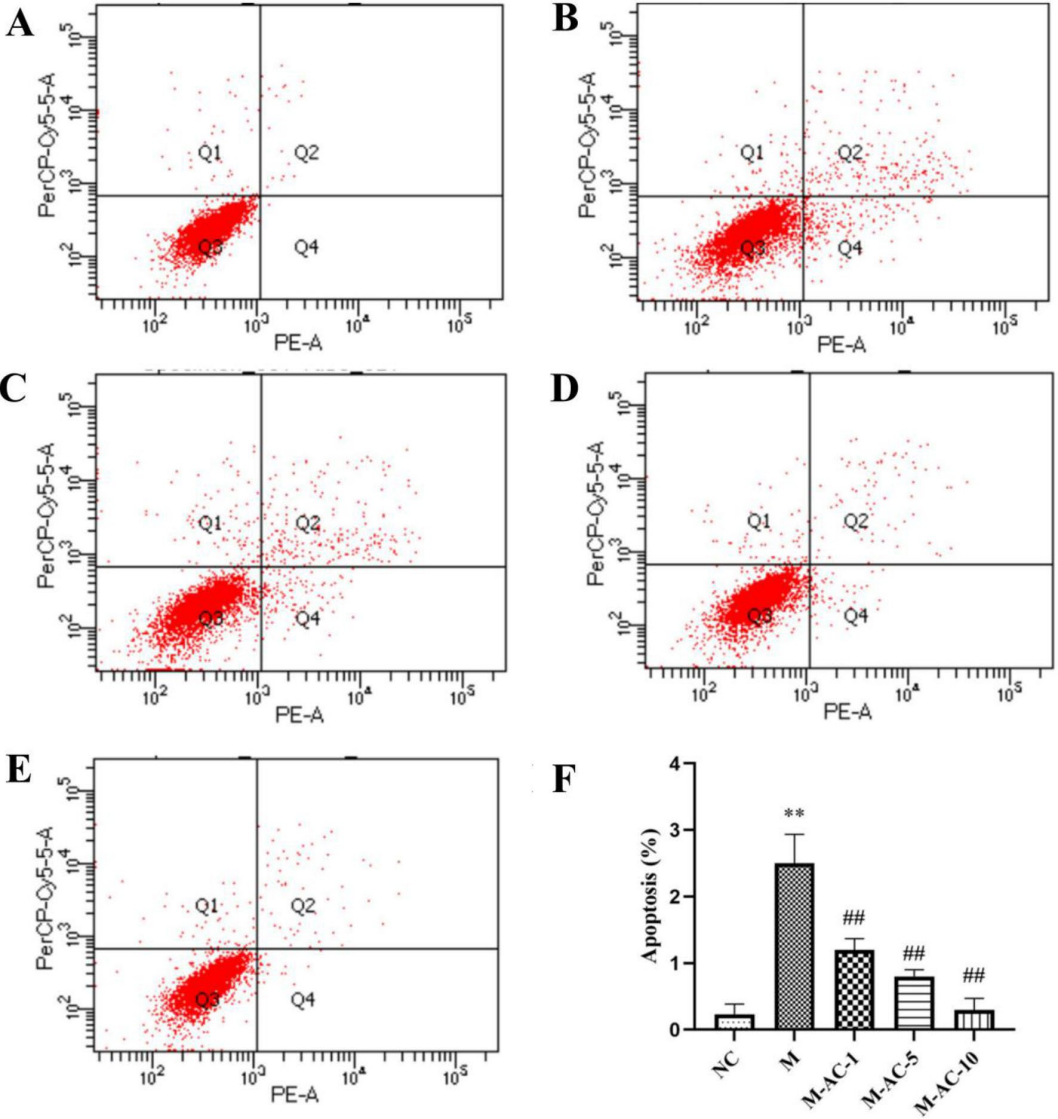
Effect of acacetin on apoptosis of HUVEC cells. Flow cytometry was used to detect the apoptosis of HUVEC cells. **a** NC group, **b** M group, **c** M-AC-1 group, **d** M-AC-5 group, **e** M-AC-10 group, **f** the quantification results of the proportion of cells in Q4 to total cells. Values are expressed as the mean ± SD of 3 samples. ^#^*P* < 0.05, ^##^*P* < 0.01 vs. M group. ^*^P < 0.05, ^**^P < 0.01 vs. NC group

Compared with HUVEC cells in the NC group, the proportion of Q4 in the M group was significantly increased (*P* < 0.01), suggesting that the level of early apoptosis in the model group was high. The Q4 region ratio was dramatically reduced in the treatment group, manifesting that acacetin could improve the level of early dying of HUVEC cells. The group with high dose of acacetin (M-AC-10) had the best effect, so in the follow-up experiment, the drug group was given 10 μm/L of acacetin (Fig. 11).

### Effects of acacetin on inflammation in HUVEC cells

**Fig. 12:**
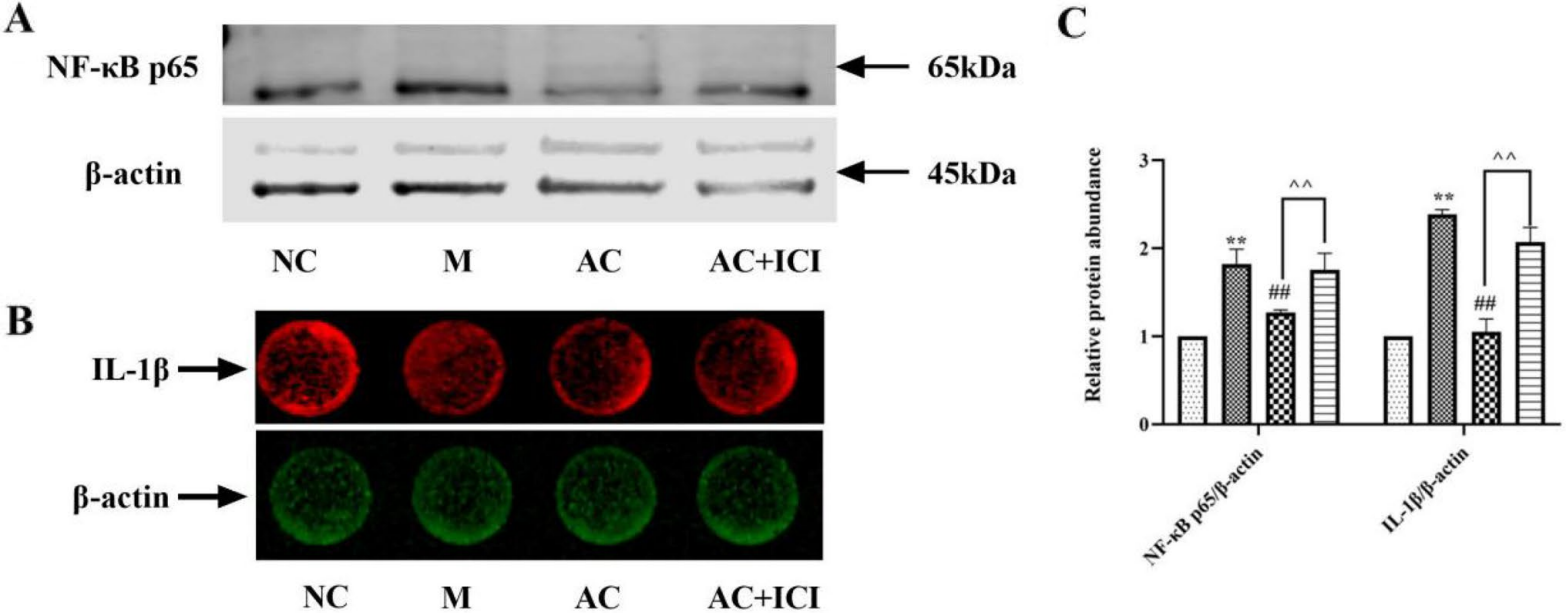
Effects of acacetin on inflammation in HUVEC cells. **a** NF-κB p65 in HUVEC cells were determined by Western Blotting. **b** IL-1β in HUVEC cells were determined by In-cell-western. **c** The quantified results of A and B. Values are expressed as the mean ± SD of 3 samples. ^#^*P* < 0.05, ^##^*P* < 0.01 vs. M group. ^*^P < 0.05, ^**^P < 0.01 vs. NC group. ^^^*P* < 0.05, ^^^^*P* < 0.01 vs. AC group

Compared with HUVEC cells in the NC group, NF-κB p65 and IL-1β in the M group were significantly increased (*P* < 0.01), suggesting an inflammatory response in the model group. Significantly decreased after administration (*P* < 0.01), suggesting that acacetin can reduce the level of cell inflammation. The trend of decreased expression of inflammatory factors was increased due to the antagonistic effect of ICI (*P* < 0.05 or 0.01), indicating that acacetin can reduce cellular inflammation by activating estrogen receptors (Fig. 12).

### Effects of acacetin on related indicators of vasodilatory function in HUVEC cells

**Fig. 13:**
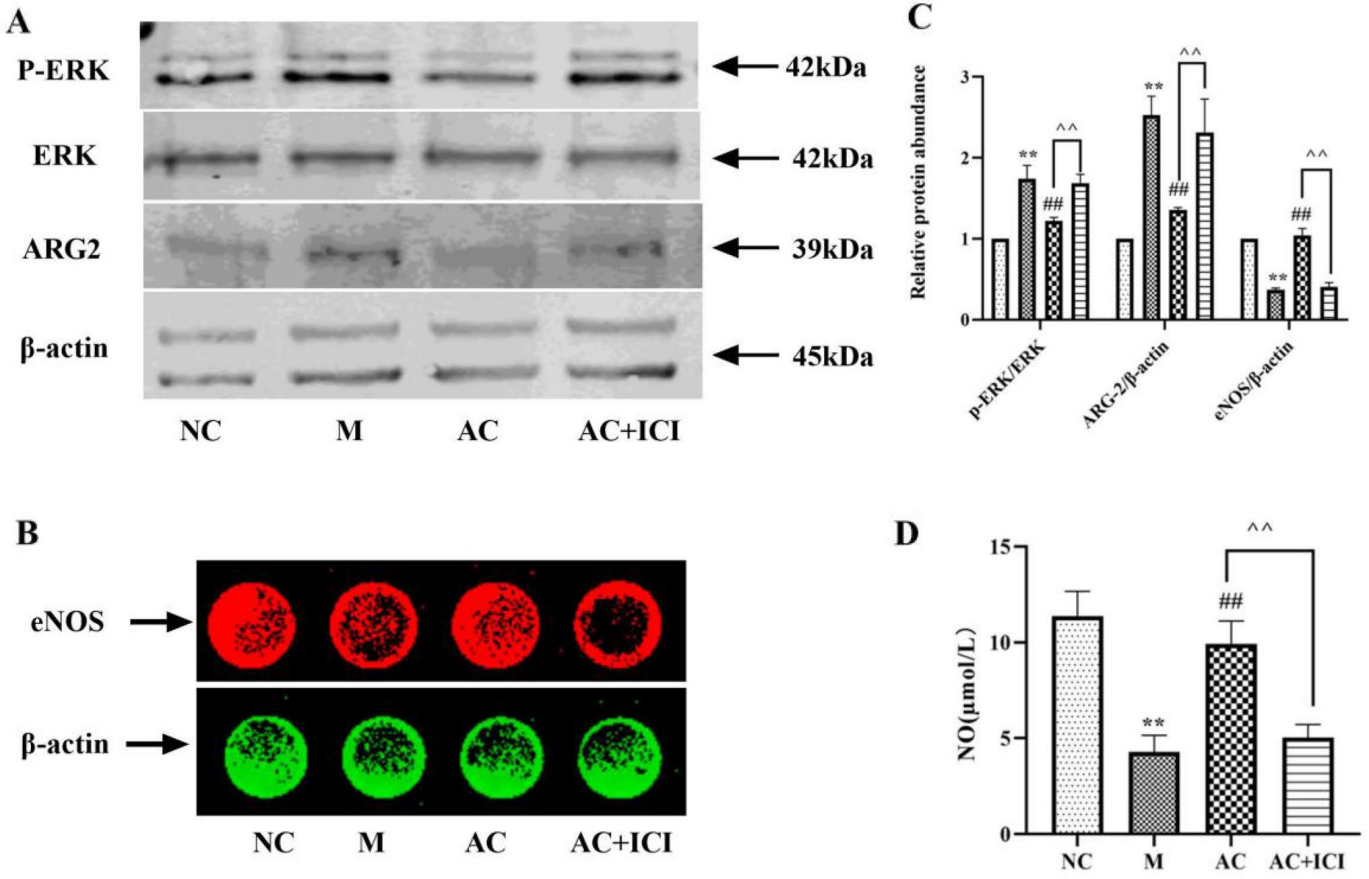
Effects of acacetin on related indicators of vasodilatory function in HUVEC cells. **a** p-ERK/ERK and ARG2 in HUVEC cells were determined by Western Blotting. **b** eNOS in HUVEC cells were determined by In-cell-western. **c** The quantified results of A and B. **d** Nitric acid reductase was used to detect NO. Values are expressed as the mean ± SD of 3 samples. ^#^*P* < 0.05, ^##^*P* < 0.01 vs. M group. ^*^P < 0.05, ^**^P < 0.01 vs. NC group. ^^^*P* < 0.05, ^^^^*P* < 0.01 vs. AC group

Compared with HUVEC cells in the NC group, p-ERK /ERK and ARG-2 in the M group were significantly increased (*P* < 0.01), and eNOS and NO were dramatically decreased (*P* < 0.05 or 0.01), suggesting impaired vasodilation in the model group. After administration, the level of related indicators in the AC group were markedly adjusted (*P* < 0.05 or 0.01), manifesting that acacetin could improve vasodilation function by promoting the production of NO. This trend was weakened by the antagonistic effect of ICI (*P* < 0.05 or 0.01), suggesting that acacetin can stimulate the production of NO by activating estrogen receptors and improves vasodilation (Fig. 13).

### Effects of acacetin on vascular fibrosis in HUVEC cells

**Fig. 14:**
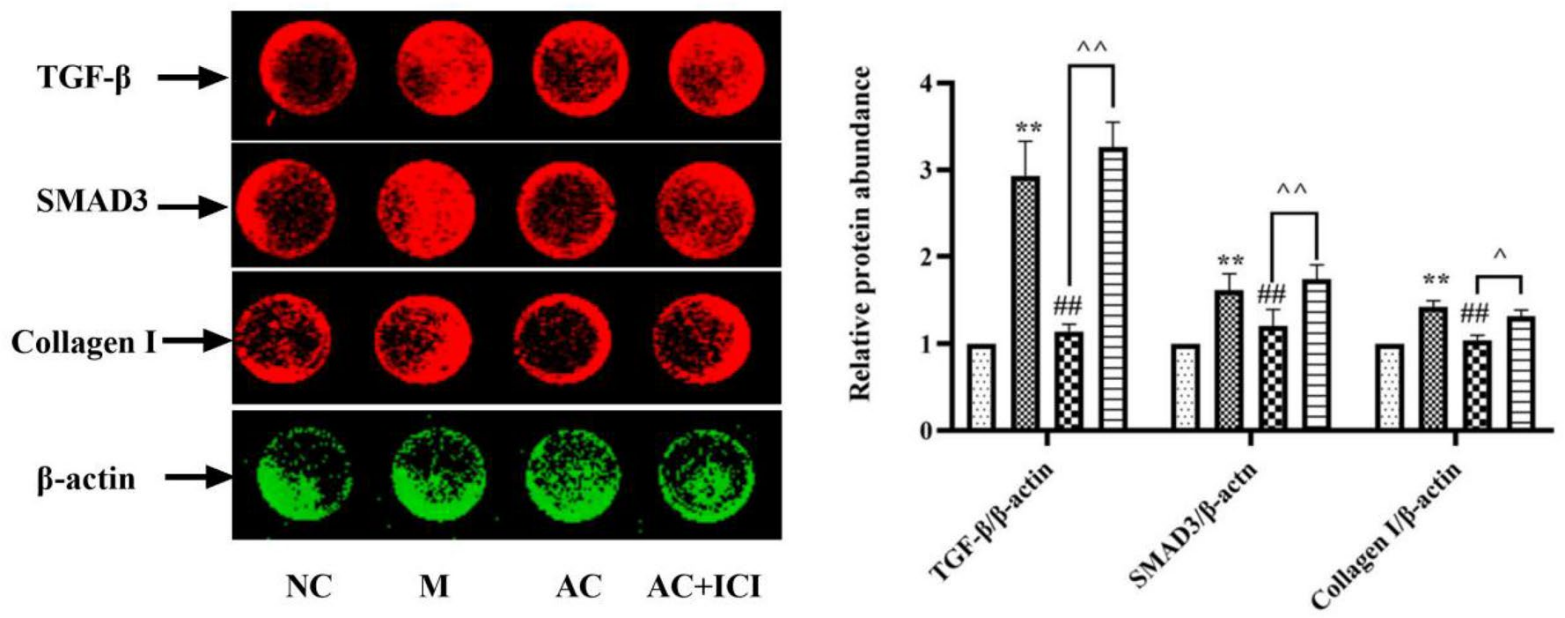
Effects of acacetin on vascular fibrosis in HUVEC cells. TGF-β, Smad3 and Collagen I in HUVEC cells were determined by In-cell-western. Values are expressed as the mean ± SD of 3 samples. ^#^*P* < 0.05, ^##^*P* < 0.01 vs. M group. ^*^P < 0.05, ^**^P < 0.01 vs. NC group. ^^^*P* < 0.05, ^^^^*P* < 0.01 vs. AC group

Compared with HUVEC cells in the NC group, the level of TGF-β, Smad3 and Collagen I in the M group were significantly increased (*P* < 0.01), suggesting the presence of fibrosis in the model group. After administration, the level of all three decreased significantly (*P* < 0.01), suggesting that acacetin can improve fibrosis. After administration, the level of all three decreased signally (*P* < 0.01), indicating that acacetin can improve fibrosis. This trend of decreased expression was increased by the antagonistic effect of ICI (*P* < 0.05 or 0.01). It is suggesting that acacetin can improve fibrosis by activating estrogen receptor (Fig. 14).

## Discussion

A large amount of clinical and epidemiological evidence indicates that hypertension is one of the major risk factors for cardiovascular disease, characterized by impaired vascular endothelial function, resulting in changes in vascular structure and function. On the other hand, insulin resistance also leads to endothelial dysfunction and other vascular complications [26, 27].Vascular endothelial injury starts at the early stage of hypertension with insulin resistance, so the prevention and improvement of vascular endothelial dysfunction is of great clinical significance for reducing the incidence of cardiovascular diseases and protecting target organs. When endothelial function is impaired, endothelial cells cannot regulate vascular homeostasis and vascular endothelial physiological balance is broken, presenting inflammatory response, vascular constriction, fibrosis and other pathological states.

In this study, after feeding 10% fructose to SHR for 7 weeks, SBP and HOMA-IR were significantly increased. HE staining results showed that the aortic wall of the rats was markedly thickened, and smooth muscle cells and endothelial cells proliferated and arranged in disorder, indicating the pathological injury of the aorta. vWF and P-Selectin are markers of endothelial dysfunction. When aortic endothelial function is damaged, endothelial cells will promote the synthesis of vWF and is secreted by Weibel-Palade bodies [28]. vWF promotes the expression of P-Selectin in vascular endothelial cells. P-Selectin is an important cell adhesion molecule, which mediates the adhesion between endothelial cells and white blood cells and promotes inflammation and thrombosis [29]. The level of vWF was observably increased and P- selectin showed an increasing trend, indicating endothelial dysfunction in rat aorta.

Vascular endothelial cell dysfunction can lead to inflammatory response. TNF-α, the earliest and most important inflammatory mediator in the inflammatory response, can lead to infiltration of inflammatory cells, and also recruit inflammatory cells to gather at the damaged site, aggravating cell damage. TNF-α and IL-1β are synergistic in stimulating the secretion of other inflammatory factors. NF-κB is a key regulator of inflammatory processes. In the resting state, the binding of NF-κB dimer to IκB is inhibited. TNF-α and IL-1β induce IκB phosphorylation and activation of NF-κB p65 in response to inflammation. Activated NF-κB p65 enhances the transcription of TNF-α and IL-1β, amplifying the initial signal of inflammation [30]. As shown in Fig. 5, the levels of TNF-α, IL-1β, and NF-κB p65 were high, resulting in an inflammatory response.

Studies have shown that MAPK pathway plays an important role in mediating inflammatory response. There are four subgroups of MAPK. The ERK pathway is closely related to stress stimulation and inflammatory response [31]. When the body produces an inflammatory response, the ERK pathway is activated. It will increase the expression of p-c-Raf and p-MEK1/2, promote the phosphorylation of ERK and further lead to the expression of p-P90RSK and p-MSK1. As an extracellular signaling kinase, ERK can promote phosphorylation of insulin receptor substrate at Ser612, which leads to insulin resistance. The expression of (Ser612) p-IRS-1 in the aorta of the model group was significantly increased. These indicators were significantly reduced after acacetin treatment, indicating that acacetin may inhibit ERK pathway and insulin resistance in SHR by reducing inflammatory response.

Studies have shown that thrombin regulatory protein (TM) can activate arginase and promote the expression of arginase [32]. However, this experimental model did not promote the secretion of ARG2 by promoting the expression of TM. TNF-α and IL-1β can up-regulate arginine expression through ERK signaling pathway [33]. ARG2 competes with eNOS for the common substrate L-arginine and eNOS is an enzyme that converts L-arginine to NO. Increased ARG2 activity resulted in decreased bioavailability of NO and impaired function of vascular endothelial cells [34]. Nostrin is a nitric oxide synthase transport factor, which has a regulatory effect on eNOS activity. It inhibits the activation of eNOS and the production of NO [35]. The results confirmed that acacetin may improve aortic endothelial dysfunction by reducing level of inflammatory factors, inhibiting the ERK pathway, down-regulating the expression of ARG2, and increasing the expression of NO.

On the other hand, arginase catalyzes the hydrolysis of L-arginine to urea and ornithine, which metabolizes polyamines by ornithine decarboxylase and proline. Polyamines promote cell proliferation and proline plays an important role in collagen synthesis, promoting vascular fibrosis and thickening [6]. Fibrosis is a pathological reaction to acute or chronic cell injury, characterized by excessive accumulation of extracellular matrix (ECM) leading to structural remodeling of various tissues and organs such as blood vessels, heart, liver, kidney and finally organ failure. TGF-β is an important regulatory factor promoting fibrosis, which can promote the transformation of fibroblasts into myofibroblasts, promote the deposition of extracellular matrix and lead to fibrosis by activating multiple signaling pathways downstream. TGF-β/Smad is one of the most classical and important signaling pathways in the pathogenesis of fibrosis. The downstream Smad3 is the intracellular kinase substrate of TGF-β receptor [36]. Collagen I, as an important part of the vascular extracellular matrix, gradually accumulated and proliferated with the progression of aortic fibrosis. As shown in Fig. 8, TGF-β/Smad3 expression level was significantly increased, Collagen I hyperplasia and disorder, Suggesting that Collagen I deposition is induced by TGF-β /Smad3 pathway in rats in the model group, which eventually led to aortic fibrosis. The inflammatory response is always accompanied by the occurrence and development of fibrosis. By down-regulating the expression of the inflammatory factors TNF-α and IL-1β, the body can effectively inhibit the expression of collagen I during fibrosis [37]. Therefore, acacetin may improve aortic fibrosis by reducing inflammatory factors. Studies have shown that asymmetric dimethyl arginine (ADMA), as an endogenous NOS inhibitor, can alter the bioavailability of NO, lead to increased blood vessel tone and microcirculation disorders in the liver and ultimately lead to liver fibrosis [38]. Therefore, acacetin may reduce inflammation, promote NO synthesis, alleviate endothelial dysfunction, reduce Collagen I expression and improve aortic fibrosis.

The occurrence of cardiovascular diseases is closely related to the level of estrogen. The estrogen can protect the vascular endothelium and vasodilation, then play the role of cardiovascular protection. The results of animal experiments showed that acacetin could improve the decreased expression of estrogen receptors in the aorta of insulin-resistant SHR rats. In order to verify whether acacia has estrogen-like effects, we adopted the uterus weight gain experiment of mice and selected female mice with immature sex and just weaned as the research objects. Mice at this age have underdeveloped ovaries, low estrogen level in the body and very sensitive to estrogen activity [39]. The mouse uterine weight gain test is the gold standard for the detection of estrogen activity. It mainly evaluates the estrogen-like activity by determining whether exogenous estrogen can promote the growth of uterus in immature female mice [13]. Phytoestrogens are compounds from natural plants that bind and activate estrogen receptors in both mammals and humans. Substances with estrogen or anti-estrogen activities are commonly called phytoestrogens. Compared with synthetic estrogens, natural phytoestrogens have lower estrogen-like activity, less side effects such as breast cancer and endometrial cancer and higher safety [40]. The results showed that acacetin had the effect of promoting uterine weight gain in mice, indicating that acacetin had estrogen activity. Moreover, it can increase the content of E2 in the serum and significantly promote the level of estrogen receptors to play the estrogen-like effect.

**Fig. 15:**
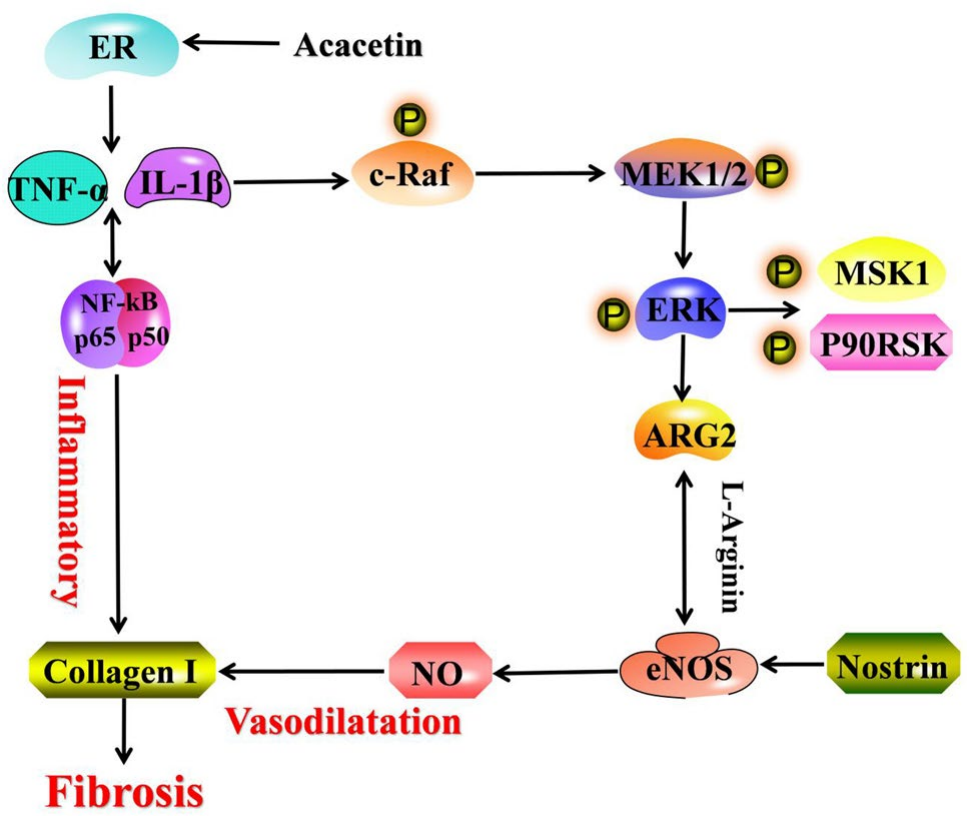
Signal pathway of improving aortic endothelial dysfunction and improve aortic fibrosis by acacetin

Estrogen can promote the release of NO, relax blood vessels and protect endothelial cells. It can also inhibit the inflammatory response of blood vessels by downregulating inflammatory factors [41, 42]. In animal experiments, acacetin was confirmed to increase the level of estrogen receptors in the aorta of insulin resistant SHR rats. Meanwhile, it was confirmed that acacetin had estrogen-like activity and could promote the expression of three estrogen receptors through the experiment of uterine weight gain in mice. Subsequently, in vitro cell experiments were conducted to investigate whether acacetin could improve endothelial dysfunction and aortic fibrosis through estrogen-like effects. To verify this thinking, we established an endothelial injury model by stimulating HUVEC cells with high glucose and salt in vitro. Cell experiments have shown that acacetin can reduce endothelial cell apoptosis, reduce inflammation, improve vascular damage and inhibit fibrosis. These improvements were antagonized by estrogen-receptor antagonists, demonstrating that acacetin is dependent on estrogen-like action to reduce inflammatory cytokines, inhibit ERK pathway, down-regulate ARG2 expression, increase the expression of NO, vasodilate, improve aortic endothelial dysfunction and improve aortic fibrosis in SHR rats with insulin-resistant. (Fig. 15).

## Conclusions

Acacetin is dependent on estrogen-like action to reduce the levels of inflammatory factors, inhibit ERK pathway, down-regulate ARG2 and increase NO to relax the blood vessels, so as to alleviate endothelial dysfunction and improve the aortic fibrosis of SHR rats with insulin-resistant.
